# Supplementary material for: Modeling glioblastoma heterogeneity as a dynamic network of cell states
Source: Mol Syst Biol. 2021 Sep 16;17(9):e10105. doi: 10.15252/msb.202010105 (PMC8444284; doi:10.15252/msb.202010105)
Supplement: Supplementary file 6 — Source Data for Figure 5 [file MSB-17-e10105-s004.zip › Figure5A_sourcedata/GSEA_3071/hallmarks_stateV.GseaPreranked.1623742546329/HALLMARK_TNFA_SIGNALING_VIA_NFKB.html]

Details for gene set HALLMARK\_TNFA\_SIGNALING\_VIA\_NFKB[GSEA]

|  || Dataset | state43071 |
| Phenotype | NoPhenotypeAvailable |
| Upregulated in class | na\_neg |
| GeneSet | HALLMARK\_TNFA\_SIGNALING\_VIA\_NFKB |
| Enrichment Score (ES) | -0.4881682 |
| Normalized Enrichment Score (NES) | -2.691828 |
| Nominal p-value | 0.0 |
| FDR q-value | 0.0 |
| FWER p-Value | 0.0 |
Table: GSEA Results Summary

  

Fig 1: Enrichment plot: HALLMARK\_TNFA\_SIGNALING\_VIA\_NFKB      
 Profile of the Running ES Score & Positions of GeneSet Members on the Rank Ordered List

  

| PROBE | GENE SYMBOL | GENE\_TITLE | RANK IN GENE LIST | RANK METRIC SCORE | RUNNING ES | CORE ENRICHMENT || 1 | BTG3 |  |  | 226 | 1.141 | -0.0263 | No |
| 2 | TNC |  |  | 246 | 1.123 | -0.0130 | No |
| 3 | FJX1 |  |  | 308 | 1.061 | -0.0088 | No |
| 4 | PLPP3 |  |  | 323 | 1.042 | 0.0042 | No |
| 5 | CDKN1A |  |  | 363 | 0.993 | 0.0116 | No |
| 6 | LITAF |  |  | 412 | 0.939 | 0.0165 | No |
| 7 | NINJ1 |  |  | 695 | 0.748 | -0.0265 | No |
| 8 | PMEPA1 |  |  | 718 | 0.740 | -0.0196 | No |
| 9 | TGIF1 |  |  | 840 | 0.683 | -0.0326 | No |
| 10 | VEGFA |  |  | 926 | 0.649 | -0.0391 | No |
| 11 | LAMB3 |  |  | 939 | 0.646 | -0.0317 | No |
| 12 | G0S2 |  |  | 1020 | 0.615 | -0.0379 | No |
| 13 | CFLAR |  |  | 1255 | 0.544 | -0.0746 | No |
| 14 | SNN |  |  | 1360 | 0.517 | -0.0869 | No |
| 15 | IRS2 |  |  | 1400 | 0.505 | -0.0867 | No |
| 16 | ZBTB10 |  |  | 1585 | 0.463 | -0.1151 | No |
| 17 | B4GALT5 |  |  | 1629 | 0.453 | -0.1166 | No |
| 18 | PDE4B |  |  | 1660 | 0.444 | -0.1157 | No |
| 19 | EGR1 |  |  | 1691 | 0.438 | -0.1149 | No |
| 20 | IER2 |  |  | 1742 | 0.428 | -0.1180 | No |
| 21 | CCNL1 |  |  | 1760 | 0.425 | -0.1149 | No |
| 22 | IL7R |  |  | 1761 | 0.425 | -0.1085 | No |
| 23 | PLAU |  |  | 1863 | 0.408 | -0.1218 | No |
| 24 | DENND5A |  |  | 2038 | 0.382 | -0.1495 | No |
| 25 | ID2 |  |  | 2104 | 0.373 | -0.1564 | No |
| 26 | BCL6 |  |  | 2130 | 0.370 | -0.1556 | No |
| 27 | FOS |  |  | 2529 | 0.319 | -0.2273 | No |
| 28 | TIPARP |  |  | 2768 | 0.292 | -0.2686 | No |
| 29 | DUSP4 |  |  | 2799 | 0.289 | -0.2700 | No |
| 30 | TSC22D1 |  |  | 2978 | 0.272 | -0.3001 | No |
| 31 | PTPRE |  |  | 3014 | 0.267 | -0.3029 | No |
| 32 | IL6ST |  |  | 3233 | -0.257 | -0.3409 | No |
| 33 | TNFAIP2 |  |  | 3314 | -0.269 | -0.3522 | No |
| 34 | BIRC3 |  |  | 3315 | -0.270 | -0.3481 | No |
| 35 | NFAT5 |  |  | 3373 | -0.278 | -0.3549 | No |
| 36 | KLF10 |  |  | 3555 | -0.308 | -0.3851 | No |
| 37 | BHLHE40 |  |  | 3620 | -0.318 | -0.3926 | No |
| 38 | YRDC |  |  | 3642 | -0.321 | -0.3918 | No |
| 39 | TNFAIP3 |  |  | 3691 | -0.330 | -0.3961 | No |
| 40 | NFIL3 |  |  | 3700 | -0.331 | -0.3926 | No |
| 41 | TRIB1 |  |  | 3821 | -0.353 | -0.4104 | No |
| 42 | MAFF |  |  | 3849 | -0.359 | -0.4101 | No |
| 43 | KLF4 |  |  | 3866 | -0.361 | -0.4078 | No |
| 44 | RIPK2 |  |  | 3867 | -0.362 | -0.4024 | No |
| 45 | TNIP2 |  |  | 3992 | -0.388 | -0.4204 | No |
| 46 | JUNB |  |  | 4027 | -0.394 | -0.4210 | No |
| 47 | ATP2B1 |  |  | 4126 | -0.416 | -0.4335 | No |
| 48 | JUN |  |  | 4135 | -0.418 | -0.4288 | No |
| 49 | SOCS3 |  |  | 4191 | -0.435 | -0.4328 | No |
| 50 | MAP2K3 |  |  | 4216 | -0.445 | -0.4307 | No |
| 51 | TANK |  |  | 4272 | -0.466 | -0.4343 | No |
| 52 | NFKB1 |  |  | 4313 | -0.476 | -0.4348 | No |
| 53 | ABCA1 |  |  | 4333 | -0.483 | -0.4312 | No |
| 54 | GADD45A |  |  | 4399 | -0.500 | -0.4362 | No |
| 55 | KDM6B |  |  | 4432 | -0.513 | -0.4346 | No |
| 56 | EHD1 |  |  | 4533 | -0.555 | -0.4455 | No |
| 57 | TNIP1 |  |  | 4756 | -0.682 | -0.4779 | Yes |
| 58 | TNFAIP6 |  |  | 4764 | -0.688 | -0.4689 | Yes |
| 59 | DUSP5 |  |  | 4796 | -0.707 | -0.4642 | Yes |
| 60 | PNRC1 |  |  | 4813 | -0.726 | -0.4564 | Yes |
| 61 | RHOB |  |  | 4820 | -0.731 | -0.4466 | Yes |
| 62 | HBEGF |  |  | 4822 | -0.734 | -0.4357 | Yes |
| 63 | DNAJB4 |  |  | 4833 | -0.739 | -0.4266 | Yes |
| 64 | SPSB1 |  |  | 4836 | -0.744 | -0.4158 | Yes |
| 65 | GFPT2 |  |  | 4873 | -0.780 | -0.4109 | Yes |
| 66 | EIF1 |  |  | 4878 | -0.785 | -0.3999 | Yes |
| 67 | LIF |  |  | 4882 | -0.789 | -0.3886 | Yes |
| 68 | NAMPT |  |  | 4906 | -0.820 | -0.3807 | Yes |
| 69 | PDLIM5 |  |  | 4907 | -0.820 | -0.3684 | Yes |
| 70 | KLF6 |  |  | 4933 | -0.849 | -0.3605 | Yes |
| 71 | SLC2A3 |  |  | 4942 | -0.859 | -0.3491 | Yes |
| 72 | PPP1R15A |  |  | 4966 | -0.882 | -0.3402 | Yes |
| 73 | SPHK1 |  |  | 4997 | -0.928 | -0.3321 | Yes |
| 74 | TNFAIP8 |  |  | 5009 | -0.949 | -0.3199 | Yes |
| 75 | MYC |  |  | 5020 | -0.972 | -0.3072 | Yes |
| 76 | EDN1 |  |  | 5038 | -1.014 | -0.2952 | Yes |
| 77 | ACKR3 |  |  | 5065 | -1.065 | -0.2842 | Yes |
| 78 | CEBPB |  |  | 5076 | -1.088 | -0.2698 | Yes |
| 79 | KLF2 |  |  | 5083 | -1.110 | -0.2543 | Yes |
| 80 | SQSTM1 |  |  | 5099 | -1.149 | -0.2399 | Yes |
| 81 | IRF1 |  |  | 5100 | -1.155 | -0.2225 | Yes |
| 82 | DUSP1 |  |  | 5138 | -1.269 | -0.2106 | Yes |
| 83 | GADD45B |  |  | 5155 | -1.335 | -0.1936 | Yes |
| 84 | SERPINE1 |  |  | 5161 | -1.377 | -0.1738 | Yes |
| 85 | PHLDA2 |  |  | 5163 | -1.384 | -0.1532 | Yes |
| 86 | PLAUR |  |  | 5185 | -1.479 | -0.1350 | Yes |
| 87 | ETS2 |  |  | 5221 | -1.671 | -0.1166 | Yes |
| 88 | IL18 |  |  | 5237 | -1.816 | -0.0922 | Yes |
| 89 | F3 |  |  | 5242 | -1.927 | -0.0640 | Yes |
| 90 | PTX3 |  |  | 5262 | -2.217 | -0.0344 | Yes |
| 91 | INHBA |  |  | 5282 | -2.697 | 0.0025 | Yes |
Table: GSEA details [plain text format]

  

Fig 2: HALLMARK\_TNFA\_SIGNALING\_VIA\_NFKB: Random ES distribution      
 Gene set null distribution of ES for **HALLMARK\_TNFA\_SIGNALING\_VIA\_NFKB**
